# Epidemiology of Exertional Rhabdomyolysis Susceptibility in Standardbred Horses Reveals Associated Risk Factors and Underlying Enhanced Performance

**DOI:** 10.1371/journal.pone.0011594

**Published:** 2010-07-14

**Authors:** Cajsa M. Isgren, Melissa M. Upjohn, Marta Fernandez-Fuente, Claire Massey, Geoff Pollott, Kristien L. P. Verheyen, Richard J. Piercy

**Affiliations:** 1 Department of Veterinary Clinical Sciences, Royal Veterinary College, London, United Kingdom; 2 Department of Veterinary Basic Sciences, Royal Veterinary College, London, United Kingdom; University Hospital Vall d'Hebron, Spain

## Abstract

**Background:**

Exertional rhabdomyolysis syndrome is recognised in many athletic horse breeds and in recent years specific forms of the syndrome have been identified. However, although Standardbred horses are used worldwide for racing, there is a paucity of information about the epidemiological and performance-related aspects of the syndrome in this breed. The objectives of this study therefore were to determine the incidence, risk factors and performance effects of exertional rhabdomyolysis syndrome in Standardbred trotters and to compare the epidemiology and genetics of the syndrome with that in other breeds.

**Methodology/Principal Findings:**

A questionnaire-based case-control study (with analysis of online race records) was conducted following identification of horses that were determined susceptible to exertional rhabdomyolysis (based on serum biochemistry) from a total of 683 horses in 22 yards. Thirty six exertional rhabdomyolysis-susceptible horses were subsequently genotyped for the skeletal muscle glycogen synthase (*GYS1*) mutation responsible for type 1 polysaccharide storage myopathy. A total of 44 susceptible horses was reported, resulting in an annual incidence of 6.4 (95% CI 4.6–8.2%) per 100 horses. Female horses were at significantly greater risk than males (odds ratio 7.1; 95% CI 2.1–23.4; p = 0.001) and nervous horses were at a greater risk than horses with calm or average temperaments (odds ratio 7.9; 95% CI 2.3–27.0; p = 0.001). Rhabdomyolysis-susceptible cases performed better from standstill starts (p = 0.04) than controls and had a higher percentage of wins (p = 0.006). All exertional rhabdomyolysis-susceptible horses tested were negative for the R309H *GYS1* mutation.

**Conclusions/Significance:**

Exertional rhabdomyolysis syndrome in Standardbred horses has a similar incidence and risk factors to the syndrome in Thoroughbred horses. If the disorder has a genetic basis in Standardbreds, improved performance in susceptible animals may be responsible for maintenance of the disorder in the population.

## Introduction

Equine exertional rhabdomyolysis syndrome (ERS) commonly affects the athletic breeds, including Thoroughbred, Quarter Horse, Arabian, Warmblood, and Standardbred [1]–[7]. The intermittent syndrome is characterised by stiffness, muscle cramping and pain and is accompanied by mild to markedly elevated plasma activities of the muscle-derived enzymes, creatine kinase (CK) and aspartate aminotransferase (AST). Work in recent years has identified specific causes of ERS that may be either acquired or inherited [5]. In the latter category, certain forms are common within specific breeds. For example, type 1 polysaccharide storage myopathy is common in Quarter Horses but not Thoroughbreds [8], [9], whereas a disorder associated with a dominantly inherited defect in sarcoplasmic calcium regulation, known as recurrent exertional rhabdomyolysis, is reported to occur in Thoroughbreds but not Quarter Horses [10], [11].

Epidemiological studies in specific breeds have defined the risk factors and welfare and economic implications of ERS. For example, between 5 and 7% of Thoroughbreds are affected worldwide [3], [12], [13] with 2- and 3-year-old horses in training being commonly affected [14]. ERS has marked economic implications in Thoroughbreds [15] with 69% of affected animals unable to race and typically losing 5.8 training days per disease episode [16], [17]. Several epidemiological studies in Thoroughbreds reveal that young fillies and nervous horses have a greater ERS risk than other horses [3], [12], [13]. In ERS-affected Quarter Horses however, (most of which carry a gain of function missense mutation (R309H) in the skeletal muscle glycogen synthase gene (*GYS1*)) [9], no associations with sex, age or temperament were found [18].

To the authors' knowledge, there have been no epidemiological studies of ERS in Standardbred horses even though this is a popular racing breed in many countries. Despite this, an early genetic association study suggested that the disorder in Standardbred horses may have an underlying genetic basis [19]. Given that racehorses are selectively bred for performance, and if ERS in this breed is indeed genetic, it would be surprising if the trait had a deleterious influence on performance; indeed, it is conceivable that the trait may confer certain performance advantages in a similar manner to that of other genetic diseases of horses [20], resulting in inadvertent maintenance of the trait in the population.

Although isolated reports exist of Standardbred horses with polysaccharide storage myopathy [1], [21], anecdotal, histopathological [22], [23] and some *in vitro* experimental evidence [24] suggests that ERS in Standardbreds may be similar, if not identical to recurrent exertional rhabdomyolysis in Thoroughbred horses. If so, then the disorders in each breed may have the same or similar associated risk factors. As such, the aim of this study was to investigate the hypothesis that ERS in Standardbreds has similar risk factors to recurrent exertional rhabdomyolysis in Thoroughbreds. In addition, we hypothesised that Standardbred horses with ERS would perform as well as, or better than matched controls in various indices. Finally, because the genetic basis of type 1 polysaccharide storage myopathy has recently been identified, we aimed to test a group of susceptible Standardbred horses for the presence of the associated *GYS1* mutation.

## Results

### Telephone survey

Of 66 Standardbred trotting trainers licensed by the Swedish Trotting Association and contacted either via letter, email or telephone in March 2007, 57 were willing to participate in the study and were contacted again via telephone (Table 1). Nine were excluded either because they did not respond to attempts to contact them, did not want to participate or were no longer training horses. The 57 remaining trainers had 1402 Standardbred horses in training, ranging from 6 to 85 horses in each yard (median 19). There were 410 stallions (29.2%), 429 geldings (30.6%) and 563 mares (40.2%) with ages ranging from 1 to 12 years. Fifty eight out of 1402 horses were reported to have been affected by ERS in the 12-month study period and thus the trainer-reported annual incidence of ERS was 4.1 cases (95% CI 3.1–5.1) per 100 horses in training. Thirty five of 57 yards (61.4%) had at least 1 horse affected; the smallest yard reporting a case had only 9 horses in training while the largest affected yard trained 65 horses. The incidence within individual yards with at least 1 case ranged from 1.7 (95% CI 0–6.0) to 20 (95% CI 0–44.7) cases per 100 horses over 12 months. Thirty of 57 (52.6%) trainers routinely measured muscle enzymes for confirmation of diagnosis; 36 of 57 (63.2%) trainers considered ERS to be a significant problem in the Standardbred breed.

**Table 1:** General information regarding Standardbred trotters in training in 57 yards in Sweden and additional information regarding each case and control horse in 22 yards participating in case control study.

	Information obtained
**Information from yards (57) in telephone survey:**	Number of Standardbred trotters in training
	Distribution of mares, gelding and stallions
	Age range of horses in training
	Number of cases in the last 12 months (March 06 - Feb 07)
	Use of serum muscle enzyme analysis to confirm cases
	Opinion of importance of ERS to the trotting industry
**Additional information from yards (22) in case control study:**	Methods of prevention
	Feeding practices
	Training regime (distance, frequency and type of work)
	Use of daily turnout (hours)
**Case (44) and control (88) horse information:**	Name
	Sex (mare, gelding or stallion)
	Age
	Temperament (calm, average or nervous)
**Additional information regarding each episode of ERS in cases:**	Name of the diagnosing vet
	Clinical signs displayed
	Use of serum muscle enzyme analysis for confirmation of ERS
	Number of days off work following the episode
	If the horse was prevented from racing due to ERS
	Level of fitness at the time
	When the episode had occurred (before, during or after training or racing)
	Time of year it occurred (spring, summer, autumn, winter or throughout the year)
	If the horse had been rested prior to the episode (if so, for how many days)
	Number of ERS episodes within the last twelve months
	If the horse had a history of lameness
**Performance data** * **for case and control horses:**	Total winnings
	Racing status (raced/unraced)
	Number of lifetime starts
	Seasons raced
	Starts in each season
	Best time from standstill and running start
	Number of first, second and third placings
	Winning and placings' percentages
	Points

*Obtained from www.travsport.se (Swedish Trotting Association website) up to and including the end of 2007. The “points” statistic is derived from performance in the last 5 starts, where 1^st^ place gives 200, 2^nd^ gives 100, 3^rd^ gives 65, 4^th^ gives 25 and 5^th^ gives 15 points. In addition each horse is awarded 1 point for each 100 SEK earned.

### Case-control study

Out of the 35 trainers reporting at least 1 case of ERS in the previous year, 13 (37.1%) relied on clinical signs only while 22 (62.9%) routinely utilised veterinary assessment of measured serum CK and AST activities to confirm the diagnosis. All of these 22 trainers were willing to participate in the case-control study (Table 1). They trained 683 Standardbred horses: 180 stallions (26.4%), 218 geldings (31.9%) and 285 mares (41.7%) with ages ranging from 2 to 11 years (median of 5 years). The number of horses in each yard ranged from 10 to 65 with a mean of 31.

Forty four of the 683 horses from the 22 yards were affected by ERS over the 12-month period; therefore the overall trainer-reported annual incidence in this study population was 6.4 cases (95% CI 4.6–8.2) per 100 horses whilst the annual incidence in individual yards ranged from 1.7 (95% CI 0–6.0) to 20 (95% CI 0–44.7) cases per 100 horses. Eleven of the 22 yards reported more than 1 case of ERS in the previous year and 28 of the 44 (63%) affected horses had had more than 1 episode. Multiple episodes ranged from 2 to 20 (median of 2) per horse.

### History and Clinical Signs

Clinical signs included stiffness in all 44 cases, sweating in 38 (86.4%), pain or distress in 19 (43.2%), swollen or hard gluteal muscles in 17 (22.7%) and recumbency in 2 (4%). The 44 cases had had a total of 139 disease episodes (median of 2) in the last year with 135 episodes (97.5%) observed within an hour after training exercise and 2 (1.4%) episodes noticed during training. One episode was reported just prior to a race and another was observed just after finishing a race.

Fifteen of 44 affected horses (34.1%) had a history of lameness. In 26 cases (59.1%) the episodes had occurred after a period of rest, ranging from 1 to 14 days, with a median of 2; in 4 of these 26 cases (15.4%) the rest was due to another disease. Thirty three of the 44 cases (75%) were given a day or more to recover from ERS ranging from 1 to 28 days with a median of 7 training days lost per episode. Twenty two of 44 cases had been prevented from racing for between 1 and 10 weeks as a result of ERS.

Most (38 of 44; 86.4%) ERS cases were considered to be in full fitness when the episode occurred, 4 cases (9%) were in medium condition while 2 (4.5%) were reported as unfit. Sixteen of the 44 affected horses (36.4%) had episodes with no associated seasonality while 9 occurred in the spring (20.5%), 6 during the summer (13.6%), 4 in the autumn (9.1%) and 9 (20.5%) during winter months. In 1 mare, the trainer believed that ERS was associated with persistent oestrus in the spring, in another case it was believed associated with a virus, while 2 horses, both stallions, were reported to be “burnt out” when the episodes occurred. In other cases no clear predisposing factor was reported.

### Management

Although it was not possible to apply statistical analysis to training and feeding practices between groups, management was broadly similar for all horses in each yard and between yards. Trainers fed a mean of 4.5 kg (range 3–6 kg) of concentrate and 9 kg (range 6–11 kg) roughage feed per horse.

### Methods of Prevention

The 22 trainers reported various methods of preventing and managing ERS and most yards used a combination of methods (Table 2). Most commonly, changes in exercise regime were reported, including not allowing a day of rest in 14 yards and regular training in 12 yards. Many trainers (9/22) advocated increased field turnout, with 2 using almost permanent turnout. Six trainers emphasised the importance of mild exercise prior to high speed training with the use of time in the field, on a vibrating platform or on a mechanical horse walker prior to exercise.

**Table 2:** Management regimes used for prevention of ERS reported by 22 Standardbred trainers in Sweden with one or more horses with ERS in training from March 2006- February 2007.

Approach used	No of responses
No day of rest allowed	14
Regular training	12
Less concentrates fed	9
More time in field	9
Set daily routines	6
Movement before training (field, band, walker)	6
No oats fed	6
Minimise stress	5
Increased use of horse walker	4
First horse to be trained in the morning	3
Wait until CK back in normal range before train again	3
Oil supplemented in feed	3
Avoiding certain bloodlines for breeding	2
After a period of rest, gradually back into work	2
Vitamin B or Mg supplemented	2
Low dose acepromazine, when brought back into work	1
Less protein rich hay	1
Use of magnetic rugs	1

Alterations in feeding regimes were commonly employed, with 9 trainers reducing the amount of concentrates fed when rested. Six trainers ensured that affected horses had set daily routines while 5 trainers tried to minimize stress as much as possible. One trainer reported using low dose oral acepromazine when returning horses to work however no trainers used dantrolene sodium or electrolyte supplementation for prevention.

### Associated Risk Factors and Performance Indicators

Descriptive statistics of continuous variables for cases and controls are shown in Table 3. Univariable analysis indicated that female horses were 4.3 (95% CI 1.9–9.8; p<0.001) times more likely to have ERS than male horses, and nervous horses were 5.5 (95% CI 2.6–12.0; p<0.001) times more likely to be affected compared to those of calm or average temperament. There was no significant association between age and ERS (p = 0.69). Of the performance indicators investigated, only time from standstill start, winning percentage and placing percentage were significantly associated with ERS status: ERS horses were faster from standstill starts (odds ratio (OR) per second increase = 0.7; 95% CI 0.5–0.9; p = 0.01) and had higher winning (OR = 1.04; 95% CI 1.00–1.08; p = 0.04) and placing percentage than control horses (OR = 1.03; 95% CI 1.00–1.06; p = 0.03). Other variables that were eligible for consideration in a multivariable model were whether or not the horse was unraced (p = 0.10), prize money won per year of age of the horse (p = 0.06) and number of seasons raced per year of age (p = 0.15).

**Table 3:** Descriptive statistics of continuous horse-level and performance variables for Swedish Standardbred trotters with (cases) and without (controls) exertional rhabdomyolysis syndrome.

Variable		Cases (n = 44)			Controls (n = 88)	
	Mean (sd)	Median (iqr)	Range	Mean (sd)	Median (iqr)	Range
Age	5.2 (2.1)	5 (4–6)	2–11	5.4 (2.1)	5 (4–7)	2–11
Time from standstill start (seconds/km)	76.0 (1.1)	76.2 (75.4–76.6)	73.4–78.1	76.9 (2.0)	76.6 (75.7–78.1)	73.7–82.6
Time from running start (seconds/km)	74.6 (2.0)	74.1 (72.9–76.2)	71.8–78.9	74.7 (1.9)	74.3 (73.4–75.9)	71.4–82.8
Winning %	19.9 (16.9)	16 (10–23)	0–75	15.4 (12.4)	13 (5–26)	0–56
Placing %	42.6 (19.7)	42 (32–50)	0–100	36.9 (16.8)	38 (25–46)	0–80
Points	492.1 (506.4)	345 (50–599)	0–2010	586.9 (703.5)	385 (147–681)	0–4235
Lifetime starts	32.4 (27.7)	23.5 (14–43.5)	0–130	31.0 (24.3)	24.5 (13–44.5)	1–106
Seasons raced	3.5 (1.8)	3 (2–5)	1–9	3.4 (2.1)	3 (2–5)	1–9
Total prize money won (SEK)	368,008 (390,979)	247,000 (100,000–525,856)	13,000–2,124,923	356,411 (460,059)	188,100 (46,800–423,500)	0–2,461,302
Prize money per start (SEK)	10, 927 (8,934)	7,719 (4,548–14,674)	0–38,442	10,080 (9,953)	6,691 (3,416–13,141)	0–51,623
Prize money per start per season raced (SEK)	4,056 (4,209)	2,643 (1,228–4,945)	0–19,221	3,899 (4,657)	2,472 (1,173–4,945)	0–25,812
Prize money per season (SEK)	101,289 (87,501)	69,641 (38,500–158,605)	11,350–365,201	94,955 (104,070)	66,000 (25,600–111,250)	0– 492,260
Prize money per age of horse (SEK)	65,869 (56,874)	47,812 (21,060– 105,177)	4,333–193,175	56,089 (69,231)	30,332 (9,118–76,243)	0–351,615
Starts per season raced	8.5 (3.9)	9 (5.8–11)	1–18.6	8.8 (3.9)	8.8 (6–11.5)	1–19.3
Seasons raced per year of age	0.6 (0.2)	0.7 (0.5–0.8)	0–1	0.5 (0.2)	0.6 (0.4–0.7)	0–0.9

sd  =  standard deviation; iqr  =  inter-quartile range.

Results of multivariable conditional logistic regression are shown in Table 4. Two final models were obtained that fit the data equally well and therefore both are presented. Sex and temperament remained significantly associated with ERS. Time from standstill start and either winning percentage or placing percentage were also associated with ERS status when adjusted for sex and temperament. When both winning percentage and placing percentage were included in the same model (with sex, temperament and time from standstill start), these variables became non-significant, likely due to their strong correlation. Other variables that were considered for inclusion in a multivariable model became non-significant when adjusted for sex and temperament.

**Table 4:** Results of multivariable conditional logistic regression analysis of variables associated with exertional rhabdomyolysis syndrome in Swedish Standardbred trotters.

Variable	Category	Adjusted Odds ratio	95% Confidence interval	P_Wald_	P_LRS_
***Model 1***					
Sex	Male	1			
	Female	7.1	2.1, 23.4	0.001	<0.001
Temperament	Calm or average	1			
	Nervous	7.9	2.3, 27.0	0.001	<0.001
Time from standstill start (sec/km)		0.6	0.4, 1.0	0.06	0.04
Winning %		1.1	1.0, 1.1	0.008	0.006
					
***Model 2***					
Sex	Male	1			
	Female	6.4	2.0, 20.5	0.002	<0.001
Temperament	Calm or average	1			
	Nervous	6.6	2.2, 20.1	0.001	<0.001
Time from standstill start (sec/km)		0.6	0.4, 1.0	0.07	0.04
Placing %		1.1	1.0, 1.1	0.003	0.001

Models were derived using a stepwise forward selection approach with variables retained in the model if they were significantly associated with ERS (P_Wald_ <0.05) and/or improved model fit (P_LRS_ <0.05). Pwald =  Wald test P-value; P_LRS_  =  Likelihood Ratio Statistic P-value.

### Genotyping

DNA samples were available from 36 of the ERS-susceptible Standardbred horses and all were negative for the R309H *GYS1* mutation.

## Discussion

This study is the first that has examined epidemiological aspects of ERS in Standardbred horses. Results indicate that approximately 6% of horses were affected by ERS per year and that being female and having a nervous temperament were significant risk factors. Common regimes used to manage ERS included alterations to diet and exercise. For certain variables, ERS-susceptible horses performed better than matched controls. Additional risk factors or associations were not found, perhaps because of the relatively low number of animals studied.

The annual incidence of ERS in the telephone study (4.1%) was lower than that reported for the case control study (6.4%) perhaps because recognition of low grade ERS was more readily achieved when trainers routinely measured CK and AST for diagnosis (i.e. the case control group). This incidence of ERS in Standardbreds is similar to that reported in other breeds: 6.7% and 4.9% in UK and USA flat racing Thoroughbreds respectively [3], [13]; 6.1% in UK National Hunt Thoroughbreds [12]; 6.7% and 9% in Polo ponies in the UK and US respectively [25]. The registered trotting population in Sweden consists of approximately 57,000 Standardbreds (www.travsport.se), hence at approximately 6% incidence, ERS in this breed likely has substantial financial and welfare implications.

Female horses were more likely to have ERS than male horses; this finding supports those in several other studies [3], [12], [13]. In Thoroughbreds, the high female predilection may represent a modifying influence on the presumed autosomal dominant inheritance [26], [27] although the lack of significant association of ERS with stages of the oestrus cycle in Thoroughbreds reduces the likelihood of a direct female sex hormone influence [28]. Standardbreds reported as having a nervous temperament were more likely to be affected by ERS than those with an average or calm temperament, similar to reports in Thoroughbreds [3], [13] and Polo ponies [25]. Female Thoroughbreds are reported to be more likely to have a nervous temperament than males [29] however, both being female and being nervous remained in each of the final multivariable models, suggesting that each is independently associated with ERS. The reason why ERS is most common in nervous horses is unknown: it could either be a consequence of the trait, or a potential cause or closely genetically linked.

Unlike some studies in Thoroughbreds where age was a significant risk factor [3], [13], no association between age and risk of ERS was found in Standardbreds. This may reflect differences in management between the two racing breeds as the majority of Standardbreds commonly enter their first race, aged 3 or 4 years rather than as 2-year-olds for flat racing Thoroughbreds. Racing career is also longer for Standardbreds (they commonly race until 10 and 12 years for females and males respectively). No association of age with ERS was found in National Hunt Thoroughbred horses (which also race at an older age) [12], or in Polo ponies [25], or in various breeds with ERS [2].

Almost 90% of Standardbreds were considered at full fitness at the time of their ERS episode. This contrasts findings in polo ponies where the majority of episodes occurred after a chukka in which the horse was perceived to be not fit enough for the level of play demanded [25]. However, all but 2 episodes were reported to be associated with training rather than racing which is similar to findings in National Hunt [12] and flat racing Thoroughbreds [3], but contrasts findings in polo ponies where competition increased the risk of ERS [25].

Performance is a multifactorial variable likely affected by training, level of fitness, genetics, behavior, temperament, experience, track position, surface conditions, coach driver and trainer. Standardbreds with ERS had a significantly faster best lap time from a standstill start than controls, and significantly more wins and/or placings. No significant difference was found between groups in fastest lap time from a running start or in any other performance indicators. Surprisingly therefore, improved performance occurs despite an underlying susceptibility to ERS: perhaps environmental or management effects on occasion create the conditions necessary for an episode of rhabdomyolysis, whereas the rest of the time, the horse is not subject to any detrimental effects; indeed there is statistically significant enhanced performance. Further studies are however required to determine the reason for the faster (standing start) lap times in the affected horses, though one conceivable explanation may be better acceleration: *in vitro* studies report that muscle from Thoroughbred horses affected by recurrent exertional rhabdomyolysis contracts and relaxes more quickly than muscle from unaffected horses [10]. Although acceleration and maximum speed have not knowingly been examined in Thoroughbreds with recurrent exertional rhabdomyolysis, enhanced performance was not a feature of susceptible Thoroughbreds in one study [3]. It is conceivable that enhanced performance characteristics (i.e. faster lap time and wins) may be responsible for the maintenance of hereditary ERS within the Standardbred population despite the welfare implications of the disorder and the time lost to training that we report.

Questionnaire-based studies have certain limitations. For example, even though trainers were encouraged to elaborate regarding disease episodes, their answers may be restricted by, and dependent on, ability to recall information and by the questions asked. It is conceivable that answers given in response to the questionnaire may have been biased by prior knowledge of the risk factors and management of the disorder in Thoroughbreds; however, in the current study we believe that this is unlikely to be a major factor since trainers were (for example) unaware of recommendations to manage ERS in Thoroughbreds with prophylactic administration of oral dantrolene [30]. Accurate disease identification may also have been affected by failing to recognise subclinical disease in control horses or by inaccurate or inconsistent interpretation of muscle enzyme results.

The epidemiological profile and risk factors identified in this study in Standardbreds are more suggestive of recurrent exertional rhabdomyolysis in Thoroughbreds than of polysaccharide storage myopathy in Quarter Horses, Warmblood or draught breeds [4], [9], [18]. Furthermore, the muscle histopathology of ERS in Standardbreds is reported to be similar to that in Thoroughbreds [23] although there are 3 cases of Standardbred horses with sarcoplasmic periodic acid schiff positive, amylase-resistant polysaccharide inclusions [1], [21], a criterion that has been used definitively to diagnose polysaccharide storage myopathy, but that may occasionally yield false positive results for the disorder now known to be associated with the R309H GYS1 mutation (type 1 polysaccharide storage myopathy) [7], [31]. In the horses examined, no ERS-susceptible animals carried this specific *GYS1* mutation [8].

It is currently unknown whether all Thoroughbreds with ERS have an identical disease, however a genetic predisposition with autosomal dominant inheritance has been proposed in Thoroughbreds in the USA [27], [32]. Given that ERS in Standardbreds is also believed to be genetic in origin [19], the similarities between the epidemiology and the histopathology of ERS in both breeds, suggests that the cause of ERS-susceptibility may be identical in both breeds, perhaps because the Standardbred breed originated from Thoroughbred lines. Consequently, management and prophylactic strategies used in Thoroughbreds may be successful in Standardbreds: although requiring further study, Standardbred horses with a susceptibility to ERS may respond favorably to oral dantrolene administration [30] and dietary modifications similar to those used in Thoroughbred horses [33], [34].

## Materials and Methods

Animal procedures in this study were conducted by licensed veterinary surgeons with informed consent in accordance with the Royal Veterinary College's ethics and welfare committee regulations.

### Telephone survey

Data were gathered using a purpose-designed form and comprised general information about horses in training (signalment, number of cases with ERS in the last 12 months (March 06 – February 07) and the procedure used to confirm the diagnosis (Table 1).

### Case-control study

Horses with trainers who reported using serum or plasma muscle enzyme activities (CK and AST) routinely to diagnose cases of ERS and who had 1 or more ERS cases in the last year were selected for inclusion in the case-control study. Of the 22 trainers who satisfied these criteria, 9 were based in southern Sweden and these were interviewed in person while the remaining 13 were spread throughout Sweden: for these trainers a telephone interview was conducted. All interviews were conducted by the same person and a purpose-designed standardised questionnaire was used for each interview (information requested is summarised in Table 1).

Trainers listed all horses considered free from ERS within their yards and subsequently, for each ERS case, 2 ERS-free horses were randomly selected (Excel; Microsoft) from the list as matched controls. For each case and control horse the age, sex and trainer-perceived temperament (nervous, average or calm) was recorded. Information about management in each yard was collected and for each case, further information regarding the episode(s) of ERS was also recorded. Performance data for case and control horses were obtained from online records (www.travsport.se); performance indicators that were assessed are listed in Table 1.

### Statistical analysis

Case-control data were analysed using conditional logistic regression, taking account of matching by trainer. Univariable analysis was conducted to estimate crude odds ratios quantifying the strength of association between horse-level and performance variables and ERS. For initial analysis, continuous variables were categorised based on quartiles to examine the shape of their association with ERS. Tests for departure from linear trend were conducted and variables modelled in continuous linear association with ERS where appropriate. Variables with a p-value of <0.20 in univariable analysis were considered for inclusion in a multivariable model to estimate adjusted odds ratios. A forward selection approach was used to build the multivariable model; variables were retained in the model if they remained significantly associated with ERS (Wald test p-value <0.05) and/or improved model fit (Likelihood Ratio Test p-value <0.05). All analyses were conducted in Stata SE 9.2 (Statacorp LLP, College Station, Texas) and the level of statistical significance was set at p<0.05.

### Genotyping

Blood samples derived from jugular venipuncture from 36 Standardbred horses identified in the study as being susceptible to ERS on the basis of routine measurement of CK and AST activity were stored in EDTA-tubes (Vacutainer) at −20C and shipped on dry ice. DNA was extracted from whole blood using a specific kit (DNeasy; Qiagen UK Ltd) and the DNA was tested for the presence of the GYS1 *R309H* mutation using the restriction fragment polymorphism assay as reported previously [8]. DNA samples from known positive and negative horses [7] were included as controls.
